# Genetic polymorphisms of *5*-*HTT* and *DAT* but not *COMT* differentially affect verbal and visuospatial working memory functioning

**DOI:** 10.1007/s00406-012-0312-0

**Published:** 2012-03-28

**Authors:** David Zilles, Jobst Meyer, Thomas Schneider-Axmann, Savira Ekawardhani, Eva Gruber, Peter Falkai, Oliver Gruber

**Affiliations:** 1 Department of Psychiatry, Center for Translational Research in Systems Neuroscience and Psychiatry, University Medical Centre, Georg August University, Von-Siebold-Str. 5, 37075 Goettingen, Germany; 2 Department of Neurobehavioral Genetics, University of Trier, Trier, Germany; 3 Department of Psychiatry and Psychotherapy, Saarland University Hospital, Homburg, Germany

**Keywords:** Genetics, Schizophrenia, Bipolar disorder, Working memory, Endophenotype, Neuroimaging

## Abstract

Working memory deficits are found in different psychiatric populations and are most pronounced in schizophrenia. There is preliminary evidence from pharmacological studies that the verbal and visuospatial subcomponents of working memory are subject to differential neurotransmitter modulation. Here, we investigated the impact of well-known polymorphisms of the dopamine transporter gene (*SLC6A3*, *DAT*) and the catechol-*O*-methyl-transferase gene (*COMT*) as well as the serotonin transporter gene (*SLC6A4*, *5*-*HTT*) on these specific working memory subcomponents in a mixed sample of patients and healthy individuals. Twenty healthy subjects and 80 patients diagnosed with schizophrenia, bipolar I disorder, or obsessive-compulsive disorder underwent genotyping for the *DAT* variable number of tandem repeats (VNTR), the *COMT* val/met-, and the *5*-*HTT* promoter length polymorphism (*5*-*HTTLPR*) and neuropsychological testing using a battery of well-characterized, brain circuit–specific working memory tasks. *DAT* genotype revealed a significant and selective effect on visuospatial working memory, while there was no effect on verbal working memory functioning. *5*-*HTT* genotype, by contrast, exerted a significant and selective effect on verbal working memory task performance. *COMT* genotype did not show any influence on either working memory domain. The results of the present study provide evidence for a differential impact of genetic polymorphisms of the dopaminergic and serotonergic systems on verbal and visuospatial working memory functioning. Together with prior evidence suggesting the existence of subgroups of schizophrenia patients exhibiting isolated deficits in only one working memory domain, this finding further supports the idea of endophenotypically and pathophysiologically distinct subgroups of schizophrenia with implications for personalized therapeutic approaches.

## Introduction

Working memory (WM) deficits have been described in different psychiatric populations and are most prominently found in schizophrenia [21]. Therefore, WM dysfunction is supposed to represent a promising endophenotype for psychotic disorders including schizophrenia and bipolar disorder [41]. According to the endophenotype concept [24], neurocognitive functions are thought to mediate between the genetic basis of psychiatric disorders and their complex and often phase-dependent clinical phenotype. Endophenotypes are assumed to be influenced more directly and (being less complex phenotypes) by a smaller number of genes compared to the disease phenotypes. The identification of genes influencing WM functioning may prove helpful to elucidate possibly shared genetic and pathophysiological processes with an impact on both endophenotype and disease. In this context, genes that directly affect the neurotransmitter systems that are presumably involved in the pathophysiology of psychiatric disorders are especially interesting. Knowledge about their pathophysiological effects could help to generate new therapeutic approaches for treating both cognitive dysfunction and clinical symptomatology.

As a basic cognitive function, WM comprises the short-term storage (maintenance) and manipulation of a limited amount of information. It is assumed to consist of different specialized subsystems including the phonological loop for the maintenance of verbal information and the visuospatial sketchpad for the maintenance of spatial information [5]. Previous functional MRI studies have identified distinct neural networks underlying specific verbal and visuospatial WM functions in healthy individuals [26, 27, 30, 32, 33, 44] and psychiatric populations [31, 37–39]. Together with complementary lesion studies [29], these data allowed to establish clear brain-behavior relationships between specific brain circuits and the verbal and visuospatial subcomponent of human WM. Behavioral deficits in patient populations can thus be attributed to specific disturbances of the underlying neurofunctional systems [28, 34, 71–73]. Interestingly, a recent study provided evidence for the existence of subgroups of patients with schizophrenia who exhibit isolated deficits in only one domain of WM [72], suggesting a selective disturbance of the neural networks underlying either verbal or visuospatial WM functioning.

In accordance with the neuroanatomical dissociation of the verbal and visuospatial WM subcomponent, there is some evidence that these WM domains are subject to differential neurotransmitter modulation. As pharmacological studies in animals have of course focused on spatial WM (for review, see [18]), particularly for this domain, an association between dopamine levels and performance has been established. Spatial WM performance is assumed to depend on an optimal level of dopaminergic signaling in terms of an inverted U-shaped curve. Most pharmacological studies in humans also investigated the effects of altered dopamine levels on the visuospatial component of WM (for reviews, see [6, 20]), while data are much scarcer for the verbal domain with negative results, for instance, for the n-back WM task [43]. Other neurotransmitter systems are also involved in the modulation of cognitive processes. Regarding serotonin, there is at least some evidence for a selective modulatory effect on verbal memory functions. One study investigating healthy adults found a worsening of verbal (digits backward) and affective (pictures of facial affect) WM performance following tryptophan loading (leading to increased cortical serotonin levels), while this condition had no effect on spatial memory span [49]. In another study, tryptophan depletion (leading to reduced serotonin synthesis) resulted in impaired delayed word recall while leaving spatial WM unaffected. Tyrosine and phenylalanine depletion (predominantly leading to dopaminergic changes), by contrast, resulted in a worse performance in spatial WM but not delayed word recall [35]. The evidence outlined above thus endorses the notion that serotonergic modulation primarily affects verbal but not visuospatial cognitive processes, whereas the opposite could be true for dopaminergic neurotransmission.

Dopamine levels are substantially regulated by its reuptake via the dopamine transporter (DAT) and by enzymatic degradation by catechol-*O*-methyl-transferase (COMT) or monoamine oxidase A (MAOA). The activity of these clearing processes varies by the existence of different polymorphisms in the respective genes (*COMT*, *MAOA*). The *COMT* valine/methionine-encoding polymorphism (Val158Met) is characterized by a decreased enzyme activity for the methionine variant at body temperature [46] and consequently higher synaptic dopamine levels. With regard to the DAT gene (*DAT*), there is a variable number of tandem repeats (VNTR) polymorphism leading to altered gene expression. Results concerning the functional consequences of the DAT VNTR polymorphism (i.e., DAT availability as measured by SPECT) have, however, been inconsistent [68].

Associations of these polymorphisms with cognitive measures have been reported. There is some evidence for better performance of individuals homozygous for the methionine-encoding variant of *COMT* compared to the val/val genotype especially in the Wisconsin card sorting test (WCST) [16, 19] and also in n-back tasks [22]. However, a recent meta-analysis [7] did not confirm these effects. Another study provides evidence for better performance of healthy *DAT* 10/10 individuals in smooth pursuit eye movement, a construct similar to visuospatial working memory [69].

Serotonin levels are in part regulated by the serotonin transporter (5-HTT). A polymorphism in the regulatory region of the respective gene (*5*-*HTT*), the *5*-*HTT* linked polymorphic region (*5*-*HTTLPR*), leads to altered *5*-*HTT* expression in vitro. Higher expression was associated with the long (L) allele as compared to the short (S) allele [36]. A positron emission tomography study found 5-HTT availability in the PFC and the parietal cortex to be associated with the performance in several verbal memory tasks including digit span forward and backward [50]. However, to our knowledge, there are no publications that reported significant associations between *5*-*HTTLPR* genotype and specific WM measures to date.

The aim of the present study was to investigate the impact of genetic polymorphisms of the dopaminergic and serotonergic system (i.e., *DAT*, *COMT*, and *5*-*HTT*) on different verbal and visuospatial WM maintenance tasks in a mixed sample of healthy probands and patients with schizophrenia, bipolar disorder, and obsessive-compulsive disorder. According to the evidence outlined above, our hypothesis was that the *DAT* and *COMT* polymorphism would impact on visuospatial but not verbal WM, whereas the 5-HTT polymorphism should vice versa influence verbal but not visuospatial WM functioning.

## Methods

### Subjects

A total of 100 subjects were included in this study comprising patients with schizophrenia (*n* = 32), bipolar I disorder (*n* = 22), obsessive-compulsive disorder (*n* = 26), and healthy individuals (*n* = 20). Selection criteria for patients were diagnosis of schizophrenia, bipolar disorder, or obsessive-compulsive disorder, according to ICD-10 criteria, and age range from 18 to 65 years. Exclusion criteria were acute suicidality, involuntary treatment, current substance abuse, history of brain trauma, diseases with alterations in cerebral metabolism, uncorrected visual or auditory disability, and mental retardation. Exclusion criteria for healthy control subjects were the same as above plus the presence of any past or present psychiatric disorder. Diagnoses were established through clinical interviews by two experienced psychiatrists using symptom checklists according to ICD-10 criteria. At the time of study participation, most patients received a stable medication including typical and atypical antipsychotics, antidepressants, mood stabilizers, and low-dose benzodiazepines depending on the disorder. For further sample characteristics, see Table 1. All subjects gave written informed consent before participation. The study was approved by the local ethics committee.

**Table 1:** Sample characteristics

	Healthy (*n* = 20)	Schizophrenia (*n* = 32)	Bipolar disorder (*n* = 22)	OCD (*n* = 26)
*Demographic factors*				
Age	34.9 (12.7)	36.3 (10.2)	43.8 (12.4)	35.5 (8.8)
Years of education	14.9 (2.66)	13.5 (3.2)	14.6 (3.1)	13.7 (2.2)
Gender (m; f)	6; 14	17; 15	11; 11	11; 15
*Genotype*				
DAT (10/10; 9-carriers)	9; 11	21; 11	14; 8	14; 12
5-HTT (L/L; other)	9; 11	11; 20 (missing: 1)	7; 15	10; 16
COMT (met/met; val/met; val/val)	4; 9; 6 (missing: 1)	6; 21; 4 (missing: 1)	6; 7; 9	6; 10; 10
*Symptom scores*				
CGI		3.94 (0.93)	3.52 (1.63)	3.85 (1.08)
MADRS		11.78 (7.54)	6.77 (7.33)	11.2 (7.42)
PANSS positive		13.83 (4.65)	8.0 (1.45)	n/a
PANSS negative		12.9 (5.05)	9.6 (4.96)	n/a
YMRS		n/a	3.38 (2.96)	n/a
Y-BOCS		n/a	n/a	20.77 (7.69)
*WM performance (accuracy in %)*				
Verbal rehearsal	94.4 (3.5)	82.9 (12.1)	89.1 (6.6)	91.4 (7.1)
Non-articulatory phonological maintenance	87.4 (5.6)	79.6 (10.8)	81.3 (9.9)	79.9 (8.2)
Visuospatial rehearsal	93.6 (7.0)	79.7 (15.1)	85.9 (11.4)	88.0 (11.6)
Visuospatial pattern maintenance	89.1 (8.8)	75.4 (13.8)	82.9 (9.8)	80.7 (13.1)

Given are the means (standard deviations) of demographic variables, genotype frequencies, and the psychopathology scores of the diagnostic groups

*CGI* Clinical Global Impression, *MADRS* Montgomery Asberg Depression Rating Scale, *PANSS* Positive and Negative Syndrome Scale, *YMRS* Young Mania Rating Scale, *Y*-*BOCS* Yale-Brown Obsessive Compulsive Scale

### Experimental design

Testing was conducted in an experimental neuropsychological laboratory under standardized conditions using a modified Sternberg paradigm (delayed match-to-sample task) [62]. The behavioral experiment consisted of four tasks testing verbal and visuospatial WM, each under single-task conditions (i.e., active rehearsal of the sample items) as well as under dual-task conditions (i.e., articulatory or visuospatial suppression). These different task conditions were conducted in a blocked manner, and each task was repeated 50 times with a short pause after 25 trials. The order of tasks was counterbalanced across subjects within each diagnostic group.

Stimulus presentation was identical for each of the four task conditions. A 5 × 5 matrix appeared on the monitor for 2 s with four squares of the matrix randomly filled by also randomly chosen, phonologically similar letters. In the 3.2-s delay period, the empty matrix was visible together with a little star moving across the screen. Simultaneously, rhythmic 4-kHz tones were presented throughout the delay period with a repetition frequency of 3.3 Hz to set the pace of the rehearsal or counting. During this interval, subjects had to keep the letters or the spatial positions in mind using the specific strategies described below. In the response phase, a single letter was presented in the matrix for 2 s. Subjects had to decide whether the probe letter (verbal WM tasks) or its position (visuospatial WM tasks) matched one of the four target letters or positions, respectively, presented at the beginning. Together with the 2-s intertrial interval, the total length of a single trial was 9.2 s resulting in a task duration of about 8 min for each of the four tasks.

In the ‘verbal rehearsal task’, subjects were instructed to vocalize the four sample letters internally one time in the presentation period and then to rehearse them throughout the delay period. In the ‘non-articulatory phonological maintenance task’, subjects were instructed to vocalize the four sample letters one time but then to continuously count from one to four during the delay period. This articulatory suppression task has been proven to selectively interfere with verbal WM [4, 33]. In the ‘visuospatial rehearsal task’, subjects were instructed to repetitively perform overt shifts of attention to the four spatial positions, a strategy termed ‘visuospatial rehearsal mechanism’ by some authors [3]. In the ‘visuospatial pattern maintenance task’, subjects had to remember the spatial pattern built by the four sample positions while performing a visuospatial suppression task by following the moving star with their eyes. This procedure had been proven to selectively interfere with visuospatial WM [4, 33].

### Genotyping

Standard PCR and genotyping were performed essentially as described earlier by our group. For DAT-specific PCR conditions, see [58]; for 5-HTTLPR, see [59]; for COMT, see [54].

### Statistical analysis

All statistical analyses were performed using PASW Statistics, version 18.0.0. According to our hypothesis regarding a differential impact of dopaminergic and serotonergic neurotransmission on verbal and visuospatial WM functions, we first performed analyses of variance (ANOVAs) for each of the three genes (*DAT*, *COMT*, and *5*-*HTT*) with genotype and diagnosis as factors. Dependent variables were visuospatial WM task performance for *DAT* and *COMT* and verbal WM task performance for *5*-*HTT*. Given the a priori hypotheses we had for each gene, adjustment for multiple comparisons was not required for these analyses. Subsequently, multivariate analyses of variance (MANOVAs) with genotype and diagnosis as factors were performed to confirm the selectivity of genotype effects on the different WM domains. When significant main effects were present, Bonferroni-corrected post hoc comparisons were made.

With regard to the genotypes, the following groups were analyzed. For *DAT*, 9-repeat carriers were grouped together as it has been established by previous studies [11, 55] because of the relatively small number of 9-repeat homozygote subjects. Group comparisons were thus made for 10-repeat homozygotes versus 9-repeat carriers. For *COMT*, there are reports on a dose-dependent effect of the met-allele on cognitive performance [19], and thus all genotypes (val/val, val/met, met/met) were considered in the primary analysis. However, as there is also evidence for a worse performance of val-homozygotes compared to met-carriers [19] and a better performance of met-homozygotes compared to val-carriers [16], additional analyses were conducted using these groupings. For *5*-*HTT*, there is evidence for higher expression levels and higher 5-HT uptake in l-homozygotes as compared to S-carriers. S/S and S/L genotypes in turn do not show large differences [25, 70]. Thus, group comparisons were made for l-homozygotes versus S-carriers. As both the *DAT* and *COMT* polymorphisms affect dopamine levels, a possible epistatic effect in terms of a *DAT* × *COMT* interaction was analyzed by another MANOVA now considering *DAT*, *COMT*, and diagnosis as factors.

Unlike in prior publications, we did not consider age and years of education as covariates. In the subgroups built according to the genetic polymorphisms, there were no significant differences with regard to age. As to education, there is good evidence that WM more likely exerts an effect on the individual’s educational level than vice versa as WM functioning has been proven to be a strong predictor of subsequent educational success [1, 2]. To further support this assumption, we conducted a mediation analysis according to Baron and Kenny [9] performing the following linear regression analyses:

1. Predictor: genotype; criterion: years of education
2. Predictor: genotype; criterion: WM performance (putative mediator)
3. Predictors: WM performance and genotype; criterion: years of education

Mediation can be assumed if there is a significant effect in 1 and 2, a significant effect for the putative mediator in 3, and a reduced effect for genotype (independent variable) in 3 as compared to 1.

## Results

The allelic distribution of all three genes was in Hardy-Weinberg equilibrium (*DAT*: *df* = 1, χ^2^ = 1.73, *p* = 0.189; *COMT*: *df* = 1, χ^2^ = 0.126, *p* = 0.722; *5*-*HTT*: *df* = 1, χ^2^ = 0.062, *p* = 0.803). Genotype groups did not differ significantly with regard to age, gender, and diagnosis frequencies (see Table 2). As to years of education, there was a statistically significant difference for *DAT* genotype and a statistical trend for *5*-*HTT* genotype.

**Table 2:** Demographic variables and WM task performance in dependence of DAT, COMT, and 5-HTT genotypes

	DAT 10/10	9-Carriers	*p*	COMT val/val	val/met	met/met	*p*	5-HTT L/L	S-carriers	*p*
*N*	58	42		29	47	22		37	62	
Age (years)^b^	38.17 (11.1)	36.46 (11.6)	0.457	38.73 (10.5)	38.74 (11.9)	33.73 (10.6)	0.188	38.22 (12.0)	37.16 (10.9)	0.652
Gender (m/f)^a^	22/36	23/19	0.095	11/18	20/27	13/9	0.292	16/21	29/33	0.733
Education (years)^b^	13.45 (2.7)	14.94 (2.9)	0.01	13.38 (2.5)	14.23 (2.9)	14.57 (3.2)	0.294	14.78 (3.0)	13.62 (2.8)	0.053
Diagnosis (healthy-schizophrenia-bipolar disorder-OCD)^a^	9-21-14-14	11-11-8-12	0.454	6-4-9-10	9-21-7-10	4-6-6-6	0.164	9-11-7-10	11-20-15-16	0.838
WM task performance (% correct)										
Verbal rehearsal^b^	88.05 (9.7)	89.78 (9.1)	0.690	89.86 (8.2)	88.37 (10.6)	87.62 (8.9)	0.728	91.48 (7.4)	87.07 (10.3)	0.056
Non-articulatory phonological maintenance^b^	81.67 (8.8)	81.53 (10.3)	0.696	80.05 (8.9)	82.16 (9.9)	82.09 (9.6)	0.479	85.92 (7.8)	79.04 (9.5)	0.001
Visuospatial rehearsal^b^	83.43 (14.1)	89.52 (10.3)	0.049	87.71 (12.5)	83.76 (13.8)	87.39 (11.5)	0.710	87.51 (10.3)	84.95 (14.4)	0.541
Visuospatial pattern maintenance^b^	80.02 (12.4)	82.78 (13.2)	0.489	80.67 (13.2)	79.89 (12.7)	83.64 (12.5)	0.555	80.84 (11.6)	81.27 (13.6)	0.649

Given are the means (standard deviations) and frequencies, respectively, together with the *p*-values of the statistical tests

^a^Pearson χ^2^

^b^ANOVA (factor genotype for age and education; factors diagnosis, and genotype for WM performance)

According to our hypothesis that WM performance would impact on the participants’ educational level rather than vice versa, a mediation analysis according to Baron and Kenny [9] was conducted to confirm the expected mediating effect of WM performance on the dependent variable ‘years of education’. According to this approach, a series of linear regression analyses revealed:

1. A significant effect of genotype on years of education (*DAT*: *p* = 0.01; *5*-*HTT*: *p* = 0.053)
2. Significant effects of *DAT* on visuospatial WM performance (visuospatial rehearsal: *p* = 0.02) and of *5*-*HTT* on verbal WM performance (verbal rehearsal: *p* = 0.024)
3. Significant effects of verbal (verbal rehearsal: *p* < 0.0005) and visuospatial WM performance (visuospatial rehearsal: *p* = 0.006) on years of education when controlling for genotype; a reduced significance of the effect of genotype on years of education (*DAT*: *p* = 0.044; *5*-*HTT*: *p* = 0.217) when controlling for the respective WM task

Thus, a mediating effect of WM performance on years of education can be assumed. Consequently, years of education was not used as a covariate in the further analyses.

According to our a priori hypotheses, we then conducted analyses of variance (factors: diagnosis and genetic polymorphism) to determine the effects of the above-described *DAT* and *COMT* polymorphisms on visuospatial WM performance and of the *5*-*HTT* polymorphism on verbal WM performance. In the following, the results are presented for each gene separately (cf. Table 2).

*DAT*: There was a significant effect of *DAT* genotype on ‘visuospatial rehearsal’ (*F* (1, 92) = 3.97, *p* = 0.049) with 10-repeat homozygote subjects showing a worse performance compared to the 9-repeat carriers. No significant effect was observed regarding ‘visuospatial pattern maintenance’.

As for diagnosis, there was a significant effect for both visuospatial WM tasks (visuospatial rehearsal: *F* (3, 92) = 5.3, *p* = 0.002; visuospatial pattern maintenance: *F* (3, 92) = 6.08, *p* = 0.001). Bonferroni-corrected pairwise comparisons revealed a significantly worse performance of the schizophrenia group when compared to the healthy subjects for both visuospatial WM tasks. There was no significant *DAT*-diagnosis interaction effect.

*COMT*: No significant effects of *COMT* genotype (val/val vs. val/met vs. met/met) on visuospatial WM performance were observed. Also when using other contrasts (i.e., val-homozygotes vs. met-homozygotes, val-carriers vs. met-homozygotes, and met-carriers vs. val-homozygotes), there was no significant effect of *COMT* genotype.

A significant effect of diagnosis was observed for both visuospatial WM tasks (visuospatial rehearsal: *F* (3, 86) = 3.23, *p* = 0.026; visuospatial pattern maintenance: *F* (3, 86) = 3.46, *p* = 0.02) with the schizophrenia group performing worse than healthy subjects in both tasks. There was no significant *COMT*-diagnosis interaction effect.

*5*-*HTT*: There was a significant effect of *5*-*HTT* genotype on ‘non-articulatory phonological maintenance’ (*F* (1, 91) = 3.74, *p* = 0.001) and a statistical trend for the ‘verbal rehearsal’ task (*F* (1, 91) = 12.13, *p* = 0.056). l-homozygote subjects performed better compared to the S-allele carriers in both tasks.

A significant effect of diagnosis was present for both verbal WM tasks (non-articulatory phonological maintenance: *F* (3, 91) = 3.11, *p* = 0.03; verbal rehearsal: *F* (3, 91) = 7.84, *p* < 0.0005) with the schizophrenia group performing worse than healthy subjects in both tasks and worse than the OCD group in the verbal rehearsal task. There was no significant *5*-*HTT*-diagnosis interaction effect.

To further test the selectivity of genotype effects as revealed by these hypothesis-driven analyses, we then conducted multivariate analyses of variance (MANOVAs; factors genotype and diagnosis) for each gene now including all applied WM tasks. In the following, we report the results of these additional multivariate analyses. The *p*-values of the post hoc multiple comparisons were Bonferroni-adjusted. For *DAT*, there was a significant main effect of diagnosis (*p* = 0.001) but not of *DAT* genotype and *DAT*-diagnosis interaction. In the univariate tests, there were no significant effects of *DAT* genotype on verbal WM performance. For *COMT*, there was a significant main effect of diagnosis (*p* = 0.003) but not of *COMT* genotype and *COMT*-diagnosis interaction. In the univariate tests, there was no significant effect of *COMT* genotype on verbal WM performance. For *5*-*HTT*, there were significant effects of diagnosis (*p* = 0.001) and *5*-*HTT* genotype (*p* = 0.005) but not of *5*-*HTT*-diagnosis interaction. In the univariate tests, there were no significant effects of *5*-*HTT* genotype on visuospatial WM performance.

Regarding a possible epistasis of the *DAT* and *COMT* genes, no significant *DAT*-*COMT* interaction effect was observed.

## Discussion

In this study, we investigated the impact of *DAT*, *COMT*, and *5*-*HTT* polymorphisms on verbal and visuospatial WM functioning. Our main findings are a significant and selective influence of the *DAT* VNTR on a visuospatial WM maintenance task and a significant and selective influence of the *5*-*HTTLPR* on verbal WM functioning. In contrast, no significant effect on WM performance was found for the *COMT* Val158Met. This finding of a differential influence of functional genetic polymorphisms of the dopaminergic and serotonergic system on verbal versus visuospatial WM subcomponents gives support to our primary hypothesis that these WM functions do not only rely on distinct neuroanatomical networks but are also subject to differential genetic and neurotransmitter modulation.

### Effects of *DAT* on working memory

Most prior studies investigating the impact of the *DAT* polymorphism on WM-related measures failed to find significant effects. Negative results have been reported for verbal fluency [55], WM n-back tasks [11, 12, 17], and WCST [17, 64]. In contrast, a significantly better performance of healthy *DAT* 10-repeat homozygotes as compared to 9-repeat carriers (and vice versa for schizophrenia patients) was found for smooth pursuit eye movement (SPEM) [69]. SPEM is a construct similar to visuospatial WM, and fMRI studies revealed overlapping brain activations during the performance of SPEM and the specific visuospatial WM task used here [10, 33]. Thus, the finding of *DAT* genotype affecting the performance in both tasks could reflect this functional and anatomical overlap. Further evidence for an effect of *DAT* genotype on spatial WM comes from an animal study, which reported Y Maze Spontaneous Alternation, a measure of spatial WM in rodents, to be impaired in *DAT* knockout mice as compared to the wild type [48]. The findings of the present study and the earlier literature outlined above thus support our hypothesis of an influence of *DAT* genotype (and thus dopamine) particularly on the visuospatial WM domain. However, interpretation of these data is restricted by different directions of the *DAT* effect. While we found a better visuospatial WM performance in 9-repeat carriers as compared to 10-repeat homozygotes in our mixed sample, Wonodi [69] reported the opposite effect with a better performance of the healthy 10-homozygotes. These conflicting results may be also due to the divergent findings regarding the effects of the *DAT* polymorphism on protein expression and consequent effects on baseline dopamine levels. While some authors reported higher *DAT* binding in 9-repeat carriers, others found the opposite (for review, see [68]). The effects of *DAT* genotype on cognition are hard to interpret as long as the functional effects of this polymorphism are not sufficiently understood. Although there is limited but consistent evidence for an influence of the *DAT* polymorphism on visuospatial WM and related brain functions, possible mechanisms at the level of dopaminergic neurotransmission remain to be elucidated.

### Effects of *COMT* on working memory

Although a recent meta-analysis [7] did not find any significant effects of the *COMT* Val158Met-encoding polymorphism on various cognitive measures, some previous studies found such effects on n-back tasks and WCST [19, 22]. In our current study, we did not observe a significant effect of *COMT* genotype on either verbal or visuospatial WM maintenance tasks. A possible explanation for this divergence is the type of task used in the different studies as the construct of WM comprises a variety of test paradigms that considerably differ with respect to their cognitive demands (maintenance, manipulation, or the additional requirement of executive functions). Thus, the different WM tasks may represent at least in part different neurocognitive functions. Consistent with this, Bruder [16] investigated the effect of *COMT* genotype on various WM measures in healthy adults and found genotype differences only for the WCST and the letter number sequencing test (LNS) but not for a spatial delayed response task, n-back task, and word serial position test. The authors argued that *COMT* could selectively affect the higher-order processing components (like executive functions and mental manipulation) but not mere maintenance and updating processes. This would be in line with the negative finding for *COMT* in our study as we also applied rather simple WM maintenance tasks.

### Differential effects of *DAT* and *COMT* on working memory

The finding of a significant influence of the *DAT* but not the *COMT* polymorphism on visuospatial WM is to some extent counterintuitive. It is widely accepted that WM functioning is particularly related to the prefrontal cortex (PFC) (e.g., [23]). Given the relative minor role of *DAT* for dopamine clearance in this area as compared to striatum [60, 61, 67], one would rather expect the *COMT* genotype to impact on WM functioning. However, the neural networks activated during WM task performance are not restricted to the PFC but involve other brain areas in which the relative importance of *DAT* may be different from PFC. (As Patricia Goldman-Rakic said, ‘understanding the prefrontal component is but one part of the grand design’ [23]). For the specific visuospatial WM task used here, functional activations have been shown in a bilateral prefrontoparietal network including, for instance, the cortices along posterior parts of the superior frontal sulcus and along the intraparietal sulcus [33]. Interestingly, there is evidence for the presence of *DAT* also apart from brain regions with established dopaminergic pathways. Lewis [47] found that *DAT*-immunoreactive axons are present throughout the cortex in macaques with a particularly high density of *DAT* immunoreactivity found in the posterior parietal cortex, suggesting a direct dopaminergic influence in this area. For humans, there is at least indirect evidence for an influence of *DAT* in the (posterior) parietal cortex. Two recent fMRI studies found that differences in both *DAT* availability [65] and *DAT* genotype [63] impact on brain activations in parietal regions. Thus, there is evidence for a relevant influence of *DAT* (be it of direct or indirect nature) on the human parietal cortex, which is activated during visuospatial WM task performance. This provides a possible explanation for our finding of a significant influence of the DAT genotype on visuospatial WM functioning.

### Effects of *5*-*HTT* on working memory

To our knowledge, this is the first report of a significant effect of the *5*-*HTTLPR* genotype on specific verbal WM functions. Studies of an association of *5*-*HTTLPR* with performance in the WCST yielded conflicting results with sometimes the S-allele [13] and sometimes l-homozygosity [14] being associated with better executive performance. Other recently published studies did not detect any associations between the *5*-*HTT* polymorphism and WM measures, specifically the Count Span task [8] and a WM summary score comprising a visuospatial WM task and LNS [45].

Our finding of a better performance of l-homozygote individuals in specific verbal WM tasks thus seems to be inconsistent with these prior results. This inconsistency may, however, be explained by the type of WM measure used in the different studies. While we applied simple WM maintenance tasks, the above-mentioned studies used more complex paradigms (including more than only WM demands) or composite scores (comprising different task demands and WM domains). However, the more complex the paradigm and the more cognitive processes are involved, the more likely various neurotransmitter systems will play a role in task performance. A lack of process specificity of the applied tasks will thus dilute existing genetic effects and lead to a decreased sensitivity for their detection. Future studies should therefore apply cognitive processes that are deconstructed as far as possible to the level of ‘cognitive atoms’ as the use of such simple and purer cognitive processes seems to be a more promising approach for the investigation of genetic effects on cognition.

### Limitations

The findings of our study are of course limited by the relatively small sample size and the heterogeneity of the study sample that comprised healthy subjects as well as patients with schizophrenia, bipolar I disorder, and obsessive-compulsive disorder. Replication is needed using larger and more homogeneous samples. This will also help to control for putative effects of psychopharmacological treatment on WM performance which we cannot exclude in our mixed and medicated patient sample. Publications on this issue, however, yielded contradictory results concerning whether and how antipsychotics [52, 56] and antidepressants [40, 57, 66] affect WM functioning with some evidence that putative effects depend more on individual differences in the receptor profiles of the substances (e.g., anticholinergic properties) than on mere substance class or dose effects [51, 53]. To solve the issue of possible medication effects in patient samples, studies including drug-naïve patients or prespecified pharmacological regimens are needed.

A further limitation is the use of the biallelic *5*-*HTTLPR* polymorphism that only considered the S- and L-allele. More recent data suggest this polymorphism to be triallelic, and only the L_A_-allele was associated with increased *5*-*HTT* mRNA levels in contrast to the L_G_-allele, which was functionally similar to the S-allele [42]. However, other recently published studies also used the biallelic *5*-*HTT* polymorphism and reported significant effects on both cognitive measures [13, 14] and *5*-*HTT* mRNA levels and activity [70]. Furthermore, a recent SPECT study investigating the effects of the *5*-*HTTLPR* polymorphism on resting state perfusion in acutely depressed subjects revealed very similar findings comparing the biallelic and triallelic approach [15], which suggests that the consequences of analyzing the triallelic instead of the biallelic polymorphism might be small.

### Conclusions

Summarized, our study provides evidence for an indeed differential influence of genetic polymorphisms of the serotonergic and dopaminergic system on the verbal and visuospatial subcomponent of WM. Together with prior evidence suggesting the existence of subgroups of schizophrenia patients with isolated deficits in only one working memory domain, this finding further supports the idea of endophenotypically and pathophysiologically distinct subgroups of schizophrenia with implications for possible differential and personalized therapeutic approaches. Methodologically, the use of preferably simple and pure cognitive processes seems to be a promising approach for the investigation of genetic associations with cognitive parameters and is recommended for future studies.
